# Interannual Survey on Polycyclic Aromatic Hydrocarbons (PAHs) in Seawater of North Nanao Bay, Ishikawa, Japan, from 2015 to 2018: Sources, Pathways and Ecological Risk Assessment

**DOI:** 10.3390/ijerph17030904

**Published:** 2020-02-01

**Authors:** Rodrigo Mundo, Tetsuya Matsunaka, Hisanori Iwai, Shouzo Ogiso, Nobuo Suzuki, Ning Tang, Kazuichi Hayakawa, Seiya Nagao

**Affiliations:** 1 Division of Material Chemistry, Graduate School of Natural Science and Technology, Kanazawa University, Kanazawa 920-1192, Japan; rodrigomundo12@gmail.com (R.M.); seiya-nagao@se.kanazawa-u.ac.jp (S.N.); 2 Low Level Radioactivity Laboratory, Institute of Nature and Environmental Technology, Kanazawa University, Nomi 923-1224, Japan; h-iwai@se.kanazawa-u.ac.jp (H.I.); hayakawa@p.kanazawa-u.ac.jp (K.H.); 3 Noto Marine Laboratory, Institute of Nature and Environmental Technology, Kanazawa University, Noto-cho 927-0553, Japan; shozoogiso@se.kanazawa-u.ac.jp (S.O.); nobuos@staff.kanazawa-u.ac.jp (N.S.); 4 Institute of Medical, Pharmaceutical and Health Science, Kanazawa University, Kanazawa 920-1192, Japan; n_tang@staff.kanazawa-u.ac.jp; 5 Institute of Nature and Environmental Technology, Kanazawa University, 920-1192, Japan

**Keywords:** polycyclic aromatic hydrocarbons, seawater, environmental organic pollutants, source identification, propagation pathways, ecological risk assessment

## Abstract

To improve the understanding of the emission sources and pathways of polycyclic aromatic hydrocarbons (PAHs) in the coastal environments of remote areas, their particulate and dissolved concentrations were analyzed on a monthly basis from 2015 to 2018 in surface waters of Nanao Bay, Japan. The concentration of the targeted 13 species of PAHs on the United States Environmental Protection Agency (USEPA) priority pollutant list in dissolved and particle phases were separately analyzed by high-performance liquid chromatography (HPLC) coupled to a fluorescence detector. Particulate and dissolved PAHs had average concentrations of 0.72 ng∙L^−1^ and 0.95 ng∙L^−1^, respectively. While most of the samples were lower than 1 ng∙L^−1^, abnormally high levels up to 10 ng∙L^−1^ were observed in the winter of 2017–2018 for particulate PAHs. Based on the isomer ratios of Flu to Flu plus Pyr, it was possible to determine that the pyrogenic loads were greater than the petrogenic loads in all but four out of 86 samples. The predominant environmental pathway for PAHs in winter was determined to be long-range atmospheric transportation fed by the East Asian winter monsoon, while for the summer, local sources were more relevant. By the risk quotients method, it was determined that PAHs in surface seawater presented a very low risk to marine life during the interannual survey.

## 1. Introduction

Polycyclic aromatic hydrocarbons (PAHs) are a family of pollutants formed by the imperfect combustion of fossil fuels and organic matter such as wood and grass; they can also naturally occur in petroleum derivates [1]. Because of their environmental hazards and health risks to humans and marine life [2,3,4,5,6], the US Environmental Protection Agency (USEPA) has listed them as priority pollutants [7,8]. Determining the environmental behavior and distribution of PAHs is important for the assessment, management and protection of wildlife health in various environments. PAHs enter marine ecosystems by several routes, of which storm-water runoff and atmospheric deposition represented the largest sources of aquatic PAH contamination in the 20th century [9]. Furthermore, oil spills, such as those caused by tank accidents, ballast and cooling water from ships directly introduce PAHs into marine environments [10]. PAHs in marine environments are diluted and transported to other regions by physical, chemical and biological means such as evaporation, currents mixing, plankton pumping, particulate sedimentation and photodegradation [11,12]. Phyto- and zooplankton also bioaccumulate PAHs [13], affecting different food chain links. Over the last two decades, our research group has been at the center of investigations regarding the environmental fate of PAHs in the Japan Sea [10,14,15]. However, the detailed study of the spatiotemporal and phase distribution of PAHs is still crucial to understanding the impacts of human activities in estuaries, not only in major fishery areas but also in buffer regions between inland-produced pollutants and the open sea. 

Recently, the release of PAHs has increased significantly in the region around southeast Asia due to rapid economic growth [16,17]. On the other hand, Japanese rural areas are expected to show the opposite tendency due to the continuous decrease in population caused by the negative balance of natality/mortality rates and the internal migrations towards bigger cities. In Anamizu town, the closest city to our sampling point, the population was 8753 in December 2015, 8492 in December 2016, 8340 in December 2017 and 8091 in December 2018 [18], showing a steady decrease of around 300 people per year. The Noto Peninsula, located in a remote rural area facing the Japanese sea, is isolated from the rapidly economic growing “Pacific Ocean” side of Japan, in which the big cities of Tokyo, Yokohama, Nagoya, Osaka, Kobe, and Hiroshima are located. As a peninsula, terrestrial traffic can also be considered to be strictly local. In the Noto Peninsula, oyster production is the second highest income source after rice cultivation. While rice cultivation takes place in summer and autumn, oyster harvesting takes place from late autumn to early spring. These two economic activities, plus a year-round emissions load from daily activities, are the local factors that affect the otherwise wild environment.

At the Noto Air Monitoring Station of Kanazawa University in the Noto Peninsula, levels of aerosol PAHs have been monitored to investigate the influences of cross-border pollutants [19], exhibiting a seasonal variation in the levels of aerosol PAHs, which are higher in winter than in summer; however, the behavior of PAHs in aquatic environments is not-well known for this or other Japanese local areas. As mentioned above, the distribution of PAHs in the sea environment must also be investigated for the preservation of ecosystems and fisheries around the local regions. In addition, with these geographical characteristics, Nanao Bay is ideal to study the local impacts on marginal sea environments. Given the importance of studying the spatiotemporal behavior of persistent organic pollutants such as PAHs in marginal seas, surface seawater samples were analyzed monthly to determine (1) particulate and dissolved PAH average concentrations and their relative compositions, and to gain a better understanding of the possible (2) emission sources and (3) environmental pathways; furthermore, (4) ensuring the sustainability of oyster production in Nanao Bay is of utmost importance to determine the ecological risks, thus PAH levels must be monitored in this remote area. 

## 2. Materials and Methods 

### 2.1. Reagents and Chemicals

Deuterated naphthalene (Nap-d8), acenaphthene (Ace-d10), phenanthrene (Phe-d10), pyrene (Pyr-d10) and benzo[a]pyrene (BaP-d12) were obtained from Wako Pure Chemical Industries, Ltd. (Osaka, Japan) at environmental analysis grade (98% purity). The internal standard mix (ISTD) for the quantitative analysis of PAHs was prepared by dissolving the five deuterated PAHs in acetonitrile in the following concentrations (μg∙mL^−1^): Nap-d8 3.461, Ace-d10 1.236, Phe-d10 2.318, Pyr-d10 0.130, BaP-d12 0.135. Supelco EPA 610 PAH Mix, which was used as the reference standard of target PAHs, was obtained from Sigma-Aldrich (USA). Dehydrated dimethyl sulfoxide, which was of gas chromatography grade, was also obtained from Wako Pure Chemical Industries, Ltd. (Osaka, Japan). Acetonitrile (purity 99.9%) and membrane disks (HLC-DISK3, pore size 0.45 μm) were obtained from Kanto Chemical Co. (Tokyo, Japan). All other reagents were obtained from Wako Pure Chemical Industries, Ltd. (Osaka, Japan) at analytical reagent grade and were used without any treatment.

Empore C18 disks (3M Company, St. Paul, MN, USA) were preconditioned by rinsing twice with 50 mL of dichloromethane, following which they were rinsed two times with methanol. The disks were stored in methanol at −20 °C until use. Prior to use in a sample filtration, the disks were rinsed with 100 mL of ultrapure water by being passed through it.

### 2.2. Seawater Sampling and Processing

**Figure 1:**
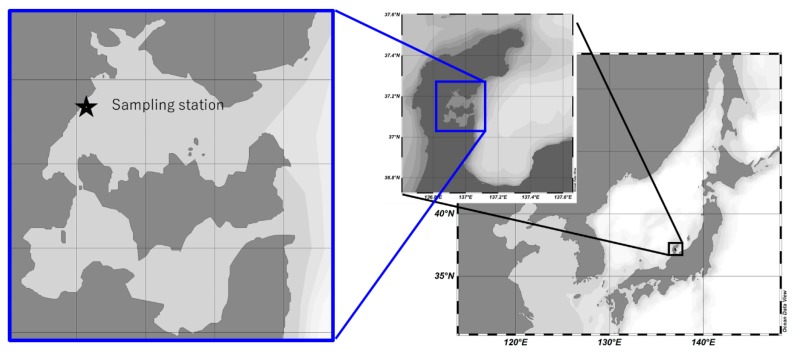
Sampling point at Noto Peninsula, Ishikawa Prefecture. July 2015–December 2018.

From 2015 to 2018, surface seawater was sampled on a monthly basis at North Nanao Bay’s most western coast (37°11′30.5″N 136°54′29.0″E) (Figure 1) and analyzed to monitor PAH. To avoid photodegradation and any residual contamination, ~10 L sea water samples were collected and stored in a polished stainless well-closed container. To collect 10 L samples considering time, and thus avoid statistical error due to point emission events, either periodic sub-sampling or continuous filtration can be considered. However, in both cases, it would be necessary to use autosamplers that ensure the preservation of sample quality; that is, to keep samples from bio- and photo-degradation, and to prevent lower-ring PAH volatilization and contamination from plastic components inside the sampler. Therefore, to overcome the relatively large uncertainty associated with single grab-samples, we opted to perform the study in a larger time span. 

Samples were passed through a GC-50 glass fiber filter (ADVANTEC, nominal pore size 0.5 μm). After adding a 100 μL aliquot of internal standard mix to the 10 L filtrate, dissolved PAHs were concentrated on an Empore C18 disk by passing the filtrate through it at a flow rate of approximately 200 mL∙min^−1^. The resulting glass fiber filter and Empore C18 disk were folded in half and dried in a desiccator over silica gel in the dark for more than three days. PAHs concentrated with the resulting glass fiber filter and Empore C18 disk were defined as particulate-phase PAHs and dissolved-phase PAHs, respectively. The amount of particle phase was estimated from the difference between the dry-weight of the glass fiber filter before and after filtration. The dried filter membranes were stored at −20 °C until extraction.

### 2.3. Extraction

Concentrated PAHs were extracted from the dried filter membrane by sonicating with two portions of 50 mL dichloromethane. To evaluate the recoveries of particulate PAHs, a 100 μL aliquot of ISTD mix was added into the glass fiber (G50) filter before the extraction process. The fragments of membranes were removed by passing the extract through a filter (ADVANTEC No. 6, Japan), and 100 μL of DMSO was added to the eluent in order to avoid the volatilization of PAHs during the later concentration process. Dichloromethane was removed using a rotary evaporator until only the DMSO remained; then, the DMSO fraction was then reconstituted to 1 mL with acetonitrile. After this, the resulting solution was filtered with a membrane disk (HLC-DISK3, pore size 0.45 μm, Kanto Chemical Co., Tokyo, Japan), and an aliquot of the solution was then injected into the HPLC for the analysis of PAHs. The extraction process was similar for dissolved PAHs; however, instead of sonication, simple vacuum filtration was utilized. 

### 2.4. HPLC Analysis

The 13 PAHs out of the 16 priority PAHs listed by the USEPA determined in the study were acenaphthene (Ace), fluorene (Fle), anthracene (Ant), fluoranthene (Flu), pyrene (Pyr), benzo[a]anthracene (BaA), chrysene (Chr), benzo[b]fluoranthene (BbF), benzo[k]fluoranthene (BkF), benzo[a]pyrene (BaP), benzo[ghi]perylene (BPe), indeno[1,2,3-cd]pyrene (IDP), and dibenzo[a,h]anthracene (DBA). The quantification and characterization of PAHs were carried out using an HPLC equipped with a fluorescence detector (FLD). The wavelength pairs of excitation and emission (Ex/Em, nm) were operated at 280/340 for Nap, Ace and Phe; 250/400 for Ant; 286/433 for Flu; 331/392 for Pyr; 264/407 for the rest of the four and five-ring PAHs; and 294/482 for the six-ring PAHs. Chromatographic separation was performed using an Inertsil ODS-P column (diameter 4.6 mm, length 250 mm, 5 μm) kept at 30 °C. The mobile phase was a mixture of acetonitrile and water operated under a gradient elution, starting at 55% and increasing to 99% acetonitrile over 65 min at a flow rate of 1 mL∙min^−1^. Accordingly, 13 PAHs were characterized by referred to the 16 PAHs in the EPA 610 Mix diluted 104 times with acetonitrile. As there were chromatographic interferences with Nap and Phe, they could not be clearly distinguished on the HPLC spectra and were thus excluded from the final analysis. The concentrations of dissolved PAH and particulate PAH were added to provide the total PAH content (total PAHs) in the marine samples. More detailed conditions were described in our previous paper [15].

### 2.5. Quality Assurance

The limit of detection (LOD) was evaluated based on a signal-to-noise ratio cutoff of 10. It ranged between 9.93 pg∙L^−1^ (Pyr) and 105.96 pg∙L^−1^ (Ace), with the correlations of linear calibration curves over 0.998. Sample blanks, as well as Millipore water blanks, were run with each batch to ensure that there was no contamination between samples. Overall recovery rates for dissolved PAH internal standards were 97.5 ± 9.62% for Phe-d10 and 86.4 ± 5.39% for Pyr-d10. Recovery rates for particulate PAH internal standards were 89.1 ± 3.49% for Phe-d10 and 94.6 ± 4.73% for Pyr-d10. To calculate the loss by extraction procedures, recoveries of Phe-d10 was utilized for Ace, Fle, Ant, and recoveries of Pyr-d10 for Flu, Pyr, BaA, Chr, BbF, BkF, BaP, DBA, BPe, and IDP. 

### 2.6. PAH Emission Source Characterization

The isomer ratios of Flu to Flu plus Pyr ([Flu]/[Flu+Pyr]) and BaA to BaA plus Chr ([BaA]/[BaA+Chr]) were analyzed to characterize the emission sources of PAHs [20]. A [Flu]/[Flu+Pyr] ratio less than 0.4 indicated the predominance of petrogenic sources, while a range between 0.4 and 0.5 suggested the dominance of fuel combustion processes, and values greater than that suggested grass, coal and wood combustion. [BaA]/[BaA+Chr] ratios smaller than 0.2 suggested petrogenic origins, values between 0.2 and 0.35 suggested automobile emissions, and those >0.35 suggested biomass combustion [20,21,22]. It must be noted that BaA presented considerably higher photo-degradation rates than Chr, which affected the source appointment by the isomer ratios method. Also, since the method was based on statistical analysis, the PAH sources which directly interfered with the studied area may not be properly reflected within the set ranges. 

### 2.7. Atmospheric Mass Long Range Transportation

To evaluate the emission origins of atmospheric PAHs, the Hybrid Single-Particle Lagrangian Integrated Trajectory (HYSPLIT) model developed by the National Oceanic and Atmospheric Administration Air Resources Laboratory, USA, was used to calculate the backward trajectory of air masses reaching the monitoring station (http://www.arl.noaa.gov/HYSPLIT_info.php) [23]. The trajectory frequency over a grid cell was calculated and then normalized by the total number of trajectories during the 32-day backward trajectories at 500 m above sea level.

### 2.8. Ecological Risk Assessment

Risk quotients (RQ) are commonly used to assess the potential ecological risk of given chemicals on aquatic organisms [24,25,26,27]. The risk levels of a given PAH are characterized by its RQ:

(1) $$RQ_{i}={\left[PAH\right]}_{i}/QV_{i}$$

where [PAH]_i_ is the concentration of the given PAH in each water sample (dissolved + particulate phase) and *QV*_i_ is the corresponding quality value. Two sets of quality values, the negligible concentrations (NCs) and the maximum permissible concentrations (MPCs), were calculated by Kalf et al. [25]. Therefore, for any given PAH, the values of *RQ_NCs__(i)_* and *RQ_MPCs(i)_* can be defined as follows:

(2) $$RQ_{NCs\left(i\right)}={\left[PAH\right]}_{i}/QV_{NCs\left(i\right)}$$

(3) $$RQ_{MPCs\left(i\right)}={\left[PAH\right]}_{i}/QV_{MPCs\left(i\right)}$$

where *RQ_NCs__(i)_* and *RQ_MPCs(i)_* represent the quality value of the NCs and MPCs for the given PAH, respectively.

Based on RQ_NCs_ and RQ_MPCs_ values, risk assignment parameters were determined [26]. Quality values for 10 of the 16 USEPA priority PAHs were originally reported [25] (Supplementary Materials). For the remaining six, we utilized toxic equivalent factors (TEF) to define the missing quality factors [27]. PAHs with similar TEFs should have similar negligible concentrations (NCs) and maximum permissible concentrations (MPCs).

To determinate the overall risk of the 13 studied PAHs, the RQ values for ∑ _(13)_ total PAHs were calculated as defined by Cao et al [28]:

(4) $$RQ_{\sum PAHs\left(NCs\right)}=\overset{13}{\underset{i=1}{\sum }}RQ_{i\left(NCs\right)}\;\;\;\;\;\;\;\left(RQ_{i\left(NCs\right)}\geq 1\right)$$

(5) $$RQ_{\sum PAHs\left(MPCs\right)}=\overset{13}{\underset{i=1}{\sum }}RQ_{i\left(MPCs\right)}\;\;\;\;\;\;\;\left(RQ_{i\left(MPCs\right)}\geq 1\right)$$

It is known that many PAH metabolites such as 6-nitrochrysene, 1,6-dinitropyrene, dibenzo[a,i]pyrene, etc., have TEFs of 10 or higher (relative to BaP) [28]. On the other hand, the presence of humic substances would reduce the biotoxicity of PAHs by adsorbing them [29]. Although these and many other factors were not considered, the method proposed by Cao et al. provided a simple method for the risk assessment based solely on the concentrations and toxic equivalent factors of each PAH, granting valuable information on the corresponding monitoring sites.

## 3. Results and Discussion 

### 3.1. General Results

**Figure 2:**
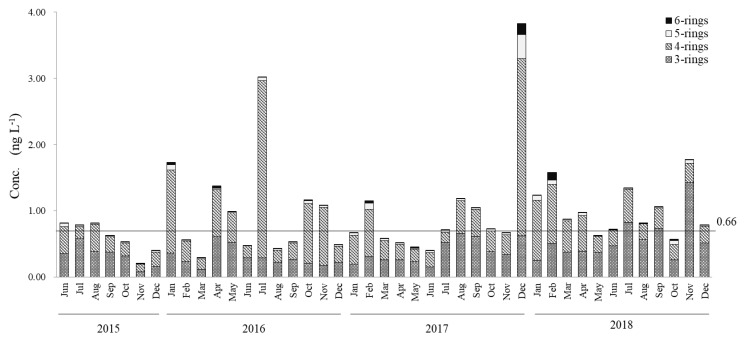
Monthly variations in concentrations of three-ring to six-ring polycyclic aromatic hydrocarbons (PAHs) in the dissolved phase in surface seawater in North Nanao Bay during 2015–2018.

**Figure 3:**
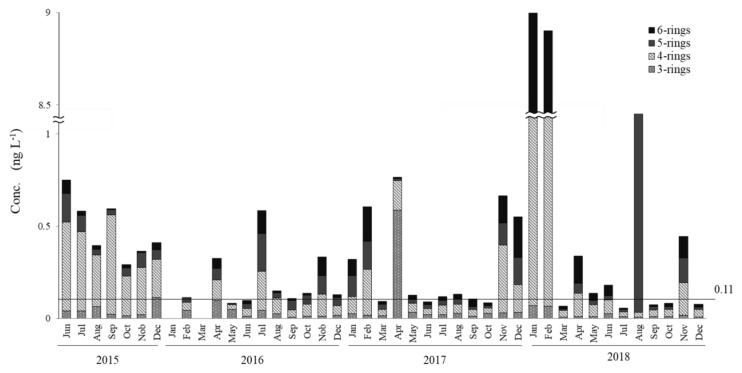
Monthly variations in concentrations of three-ring to six-ring PAHs in the particulate phase in surface seawater in North Nanao Bay during 2015–2018.

The concentrations of dissolved PAHs and particulate PAHs in the interannual PAH survey are shown in Figure 2 and Figure 3. The average concentrations for the 43-month-long survey were 0.95 ± 0.66 ng∙L^−1^ for dissolved-phase PAHs and 0.72 ± 1.88 ng∙L^−1^ for particulate PAHs. The annual averages of particulate and dissolved PAHs in 2015, 2016, 2017 and 2018 were, respectively, 0.48 ± 0.15 ng∙L^−1^ and 0.60 ± 0.22 ng∙L^−1^; 0.21 ± 0.15 ng∙L^−1^ and 1.01 ± 0.74 ng∙L^−1^; 0.30 ± 0.25 ng∙L^−1^ and 1.00 ± 0.89 ng∙L^−1^; and 1.72 ± 3.26 ng∙L^−1^ and 1.03 ± 0.36 ng∙L^−1^. The concentrations of dissolved PAHs ranged between 0.2 and 1.0 ng∙L^−1^ for most months (35 samples) except for January (1.74 ng∙L^−1^), April (1.38 ng∙L^−1^) and July (3.02 ng∙L^−1^) 2016; December 2017 (3.83 ng∙L^−1^); and January (1.24 ng∙L^−1^), February (1.58 ng∙L^−1^), July (1.34 ng∙L^−1^) and November (1.77 ng∙L^−1^) 2018 in the dissolved phase. Particulate PAHs ranged between 0.06 and 1.0 ng∙L^−1^ with January (9.06 ng∙L^−1^) and February (8.90 ng∙L^−1^) 2018 being the only samples exceeding 1.0 ng∙L^−1^. In addition, 21 out of 43 particulate PAHs samples were above 0.5 ng∙L^−1^. Total PAHs (dissolved plus particulate PAHs) ranged between 0.75 and 2.0 ng∙L^−1^ in most sampled months. If we consider only the samples below 1.0 ng∙L^−1^ of dissolved PAHs (n = 35), an average of 0.66 ± 0.22 ng∙L^−1^ is obtained, while the samples below 0.5 ng∙L^−1^ of particulate PAHs (n = 21) had an average of 0.11 ± 0.03 ng∙L^−1^. The three-ring PAHs (40%–50%) and four-ring PAHs (45%–55%) were the dominant species in dissolved PAHs. In the case of particulate PAHs, four-ring PAHs (40%−45%) and five-ring PAHs (20%−25%) were the major components. Five-ring and six-ring PAHs were distributed mainly in the particle phase, with almost neglectable amounts in the dissolved phase. In the dissolved phase, a maximum (3.83 ng∙L^−1^) was reached in December 2017 and a minimum (0.20 ng L^−1^) in November 2015. For particulate PAHs, maximums of 9.06 ng∙L^−1^ and 8.90 ng∙L^−1^ were, respectively, reached in January and February of 2018 and a minimum of 0.06 ng∙L^−1^ in July 2018.

### 3.2. Comparison of PAH Values with Other Remote Areas Worldwide

The results of this study were compared with previous data from different parts of the world. The data were selected from recent (2005~) research articles reporting PAH concentrations in surface seawater for both dissolved and particulate phases [14,30,31,32,33,34]. From the collected data, concentrations of Ace, Fle, Ant, Flu, Pyr, BaA, Chr, BbF, BkF, BaP, BPe, IDP and DBA were added and are presented in Table 1 for comparison with the results presented in this study. The average total PAH concentration at North Nanao Bay was the lowest of the collected data (1.67 ng∙L^−1^). High concentrations in and around the Mediterranean Sea (245–605 ng∙L^−1^) can be attributed to its enclosed nature and its highly populated coasts. In the case of the East China Sea (79.6 ng∙L^−1^), the high energy demand due to its rapid economic growth and highly dense population are responsible for PAH emissions, but dilution and mixing from the strong Kuroshio Current must be expected. The northwestern Japan Sea is a place known for Russian ports linked to oil extraction activities, and therefore higher pollution levels are expected (7.9 ng∙L^−1^).

### 3.3. PAHs Source Determination

**Figure 4:**
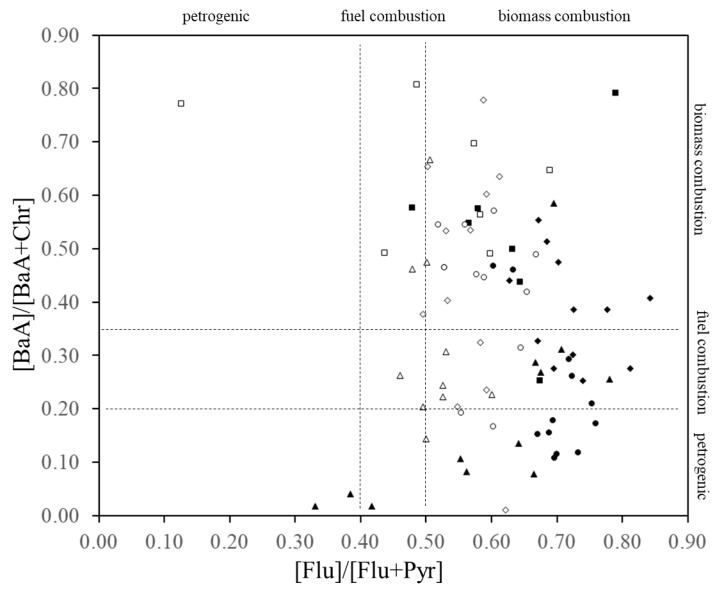
The relationship between the ratios of [BaA]/[BaA+Chr] and [Flu]/[Flu+Pyr]. Open and filled symbols represent the values of particulate and dissolved PAHs, respectively. Squares, triangles, diamonds and circles represent the data in 2015, 2016, 2017 and 2018.

As shown in Figure 4, the [Flu]/ [Flu+Pyr] ratio for most samples ranged from 0.32 to 0.85 and the [BaA]/ [BaA+Chr] ratio ranged from 0.05 to 0.80. The [Flu]/ [Flu+Pyr] ratio for the dissolved phase in surface seawater was 0.66 ± 0.11 on average, while the [BaA]/ [BaA+Chr] ratio was 0.31 ± 0.18. For particle PAHs, the ratios of [Flu]/ [Flu+Pyr] and [BaA]/ [BaA+Chr] were 0.54 ± 0.09 and 0.44 ± 0.19, respectively. The plots demonstrated that the pyrogenic inputs prevailed for most of the samples in both the dissolved and particulate phases. However, July, October and November 2016 samples had the lowest [Flu]/[Flu+Pyr] (0.33 in July, 0.38 in October, and 0.42 in November) and [BaA]/[BaA+Chr] (0.018 in July, 0.041 in October, and 0.017 in November) ratios of the survey, denoting petrogenic sources. 

### 3.4. Environmental Pathways

To identify the distribution pathways of PAHs in the North Nanao Bay, a detailed analysis of the relative distributions of the 13 components in the dissolved and particulate phases was performed. In addition, the temporal variability of each component and their relative concentrations in each phase were analyzed. 

#### 3.4.1. Long-Range Transportation of Pyrogenic PAHs

In a previous study [19], seasonal behavior was reported for all nine PAHs, presenting maximums in winter (ca. 300 pg∙m^−3^) and minimums in summer (ca.10 pg∙m^−3^) for aerosol PAHs monitored at the Noto Peninsula between 2007 to 2014. It was reported that these aerosol PAHs had pyrogenic origins [19], and the seasonality was attributed to long-range transport from Northeast China to Japan in the cold seasons and domestic sources in the warm season [19]. 

**Figure 5:**
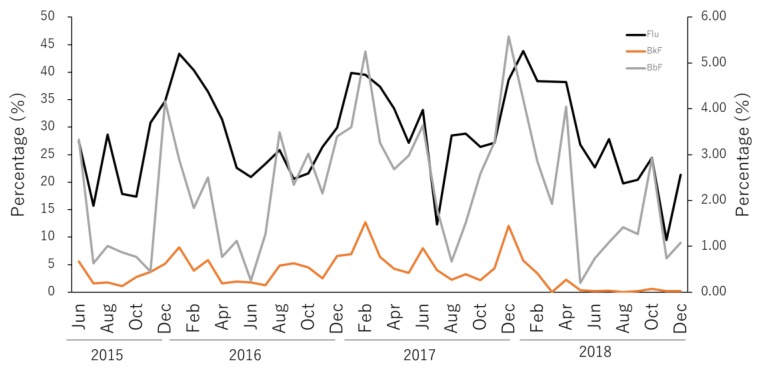
Seasonal change of relative abundance of fluoranthene (principal y-axis), benzo[b]fluoranthene (secondary y-axis) and benzo[k]fluoranthene (secondary y-axis) in the dissolved phase in North Nanao Bay during 2015–2018.

In this study, it was observed that, as a major component, the behavior of four-ring PAHs (Figure 2 and Figure 3) greatly influenced the levels of PAHs in Nanao Bay. Figure 5 shows the variation of the relative abundance of Flu, which is a four-ring PAH, in the dissolved phase. Seasonal variations were apparent in the relative compositions of Flu in dissolved-phase-surface seawater samples, with high values in winters (ca. 40%) and low values in summers (ca. 20%) (Figure 5), consistent with the previous report on aerosols [19]. Similar seasonal trends, although less clear, can be observed also in BbF and BkF. Although no reference is available for the direct derivation of BbF and BkF from Flu, their similar atomic structure and the known chemical reaction pathways for the inflamed growth of PAHs suggest that the seasonality of the relative abundance of the three compounds is linked to pyrogenic events [35,36].

**Figure 6:**
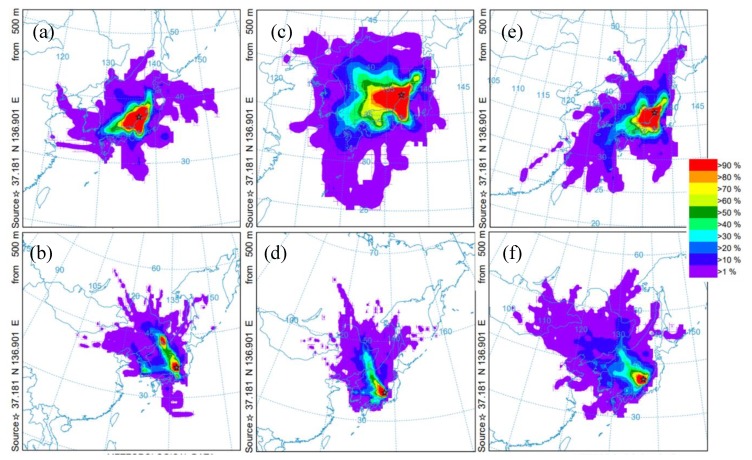
Distribution of trajectory frequencies during 32-day backward air mass (48 h) trajectories over the monitoring station. The upper panels display the summer seasons, whereas lower panels display the winter seasons. (**a**) summer, ~15 July, 2015, (**b**) winter, ~15 February, 2016, (**c**) summer, ~15 July, 2016, (**d**) winter, ~15 February, 2017, (**e**) summer, ~15 July, 2017, (**f**) winter, ~15 February, 2018.

In the other hand, relatively high levels of particle PAHs were observed in January (9.06 ng∙L^−1^) and February (8.90 ng∙L^−1^) 2018 (Figure 3). By comparing the relative abundances of the 13 measured PAHs, a common origin was confirmed. From the values of [Flu]/[Flu+Pyr] ratio (0.53 for both January and February) the pyrogenic origin of these samples was confirmed. The winter of 2017–2018 had the highest snowfalls (273 cm, including four heavy snowfall events of 44, 30, 30 and 69 cm each) during the survey (117 cm for 2016–2017, with two heavy snowfalls events of 20 and 30 cm each and 90 cm for 2015–2016, with only one heavy snowfall of 60 cm) [37]. From the backward trajectory maps, it can be seen that air masses in winter 2017/2018 came from more westerly longitudes than in the 2015/2016 and 2016/2017 winters (Figure 6), starting from east Mongolia and north China, territories known for their high concentrations of pyrogenic PAHs in winter seasons [19,38,39,40]. At the same time, the lesser influence of dry northern winds could be responsible for the larger snow falls discussed above helping to deposit the long-range transported PAHs. The higher number of high PM_10_ events registered in Seoul, Korea for the 2017/2018 winters over the same 2015/2016 and 2016/2017 seasons matches the characteristics expected for pyrogenic activities [41]. The pyrolytic sources, together with the high PM10 events, explain the high PAH levels observed solely in the particulate phase for the surface seawater samples of north Nanao Bay. Road dust washout after snow melting should be certainly considered among the local inputs; however, road dust has a petrogenic character, disagreeing with the observed data.

In conclusion, a portion of the pyrogenic emissions from East Eurasia would be transported long-range in the atmosphere to be finally deposited into the coastal aquatic environments of Japan, appearing in the seasonal trends in Flu, BbF and BkF as a percentage of dissolved PAHs, as well as contributing to the abnormally high concentrations of dissolved PAHs after the snowfalls of the winter of 2017–2018. Finally, the fact that yearly total PAH averages remained relatively constant, with 1.28 ng∙L^−1^ in 2015, 1.22 ng∙L^−1^ in 2016, 1.30 ng∙L^−1^ in 2017 and 1.75 ng∙L^−1^ in 2018, regardless of the significant population decrease (662 out of the original 8753 habitants), also supports the atmospheric long-range transportation hypothesis.

#### 3.4.2. Petrogenic Sources as Local Inputs

**Figure 7:**
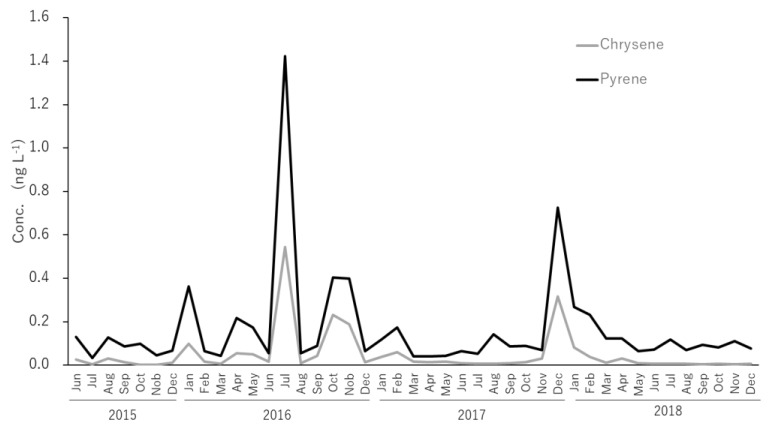
Temporal changes in the concentration of dissolved pyrene and christen in North Nanao Bay during 2015–2018.

**Figure 8:**
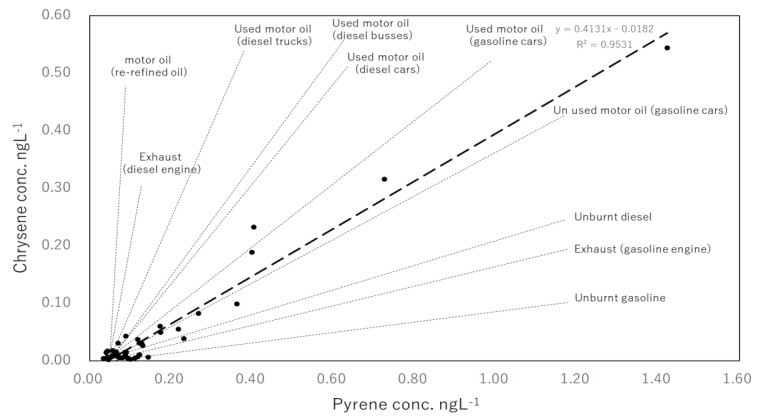
Co-variance ((r = 0.953, n = 43) of chrysene and pyrene of at the interannual survey in North Nanao Bay, Noto Peninsula.

Changes in the concentration of Pyr and Chr in dissolved PAHs are shown in Figure 7. The concentrations varied synchronously throughout the observation period. Pyr and Chr are present in all petroleum derivates; however, the ratio of Pyr/Chr varies at each product. Pyr/Chr ratios for the 43 samples were compared with previously reported Pyr/Chr ratios of known petrogenic sources (Figure 7) [42,43,44,45]. As expected from the synchronic variance shown in Figure 7, a good correlation of Pyr/Chr values were observed among the 43 samples (r^2^ = 0.953). For the known petrogenic sources, the values of [Pyr]/[Chr] ratios were larger in diesel vehicle oil compared with those in gasoline vehicles and were higher in burnt oils compared with unused oils (Figure 8). Pyr values in July, October and November 2016 were 47%, 35%, and 37%, while Chr represented 18%, 20% and 18% of dissolved PAHs, respectively. Simultaneously, as discussed in Section 3.2, the same samples had the lowest [Flu]/[Flu+Pyr] and [BaA]/[BaA+Chr] ratios of the survey, denoting petrogenic sources (Figure 4). Certainly, it may be difficult to judge when [Pyr] and [Chr] concentrations are less than 0.2 ng∙L^−1^ and 0.1 ng∙L^−1^, but in samples with higher concentrations of Pyr and Chr (i.e., July, October and November 2016, and December 2017), it can be seen that gasoline cars’ lubricating oils had the greatest influence. Clean oil for gasoline cars was the petrogenic source which presented the closest Pyr/Chr ratio (0.34) to the one observed in this study (0.41). Used oil from gasoline cars was the second (0.52) most relevant petrogenic source. Therefore, the petrogenic source found in our study can be characterized as a mixture of clean and used lubricating oil for gasoline engine vehicles. Since the discussed petrogenic sources are liquid, the lack of correlation with the particulate PAHs is to be expected. 

Therefore, it was possible to determinate that the high PAHs in the dissolved phase in July, October and November 2016, and December 2017 not only had petrogenic origins but also that the source was a mixture of clean and used lubricating oil for gasoline engine vehicles. For the rest of the samples identified as mainly having pyrogenic origins (Section 3.2), it was not possible to establish a more precise identity even of a minor—if any—petrogenic source. 

### 3.5. Ecological Risk

As expected from such low concentrations, most of the 13 PAHs had risk quotients lower than 1, representing a very low risk in North Nanao Bay for the 43 months from PAHs. However, BaA had *RQ_NCs_* > 1 in July 2015 (2.48) and December 2015 (1.08), representing a moderate risk to marine organisms. In January and February 2018, Pyr (2.06, 1.98), BaA (6.95, 6.82), BbF (2.90, 2.83), BkF (1.09, 1.07), BaP (2.64, 2.60), BPe (4.13, 4.19) and IDP (indeno[1,2,3-cd]pyrene) (2.36, 2.56) had *RQ*_NCs_ > 1 and *RQ*_MPCs_ < 1, each representing moderate risks. 

For July and December 2015, the values of $RQ_{\sum PAHs\left(NCs\right)}$ and $RQ_{\sum PAHs\left(MPCs\right)}$ were equal to RQ_NCs (BaA)_ and RQ_MPCs (BaA)_, representing an overall low risk. $RQ_{\sum PAHs\left(NCs\right)}$ values for January and February 2018 were 22.13 and 22.05, respectively; while $RQ_{\sum PAHs\left(MPCs\right)}$ was less than 1 for both months, indicating a low ecological risk. Beside December 2015, January 2018 and February 2018, the risk levels during the remaining 39 sampled months were negligible. In other scientific articles [14,30,31,32,33,34], it was observed that Phe can be considered to be a major component, especially in the dissolved phase. However, even after subtracting Nap, Phe and Acy from the cited articles in Table 1, the higher–lower relationships of total PAHs are clearly observable. For the environmental risk analysis, Phe RQs would be highly valuable information, but without trustable data, the author does not consider it appropriate to present the calculations.

## 4. Conclusions

From 2015 to 2018, particulate and dissolved PAH average concentrations and their relative compositions were analyzed with an interannual monitoring survey performed in surface waters of Nanao Bay, Japan with the aim of understanding the emission sources and environmental pathways of these pollutants in the coastal environments of remote areas. To ensure the sustainability of oyster production in Nanao Bay, the monitoring of PAH levels is of utmost importance. The concentrations of 13 USEPA priority PAHs were measured with a HPLC-fluorescence system with monthly surveys. At the end of the study, the following results were obtained:

1. Particulate and dissolved PAHs had average concentrations of 0.72 ng∙L^−1^ and 0.95 ng∙L^−1^, respectively. While most of the samples were Lower than 1 ng∙L^−1^, abnormally high levels up to ~10 ng∙L^−1^ were observed in the winter of 2017–2018 for particulate PAHs. In general, dissolved PAHs were 1.3 times greater than particulate PAHs, with the three-ring and four-ring PAHs being the dominant species in dissolved PAHs, in the range of 40%−50% and 45%−55%, while four-ring and five-ring PAH were the major components for particulate PAHs, with ranges of 45%−50% and 20%−30%, respectively. Total PAH concentrations were below 2.0 ng∙L^−1^ for most samples, showing isolated spikes in April, July and November 2016; and December 2017 and January, February 2018. The relative abundance in dissolved PAHs of Flu showed clear seasonal trends, presenting clear highs in winter (ca. 40%) and lows in summer (ca. 20%). Chr and Pyr, in dissolved PAHs, presented an overall high correlation (r^2^ = 0.95) for the 43 months of the survey.
2. Based on the isomer ratios of Flu to Flu plus Pyr, it was possible to determine that pyrogenic sources were greater than petrogenic sources in all but three samples (July, October and November 2016; dissolved PAHs). In addition, from the same isomer ratios of Flu to Flu plus Pyr, it was also visible that petrogenic sources were slightly more relevant in the particulate PAHs than in dissolved PAHs.
3. The seasonal trends in Flu as a percentage of dissolved PAHs, as well as the abnormally high concentrations of dissolved PAHs in the winter of 2017–2018, indicate that a portion of the pyrogenic emissions from East Eurasia are transported long-range in the atmosphere to be finally deposited into the coastal aquatic environments of Japan. In addition, it was determined that the high PAHs in the dissolved phase in July, October and November 2016 and December 2017 have petrogenic origins. Such petrogenic sources were further identified as a mixture of clean and used lubricating oil for gasoline engine vehicles.
4. Total PAH concentrations in surface seawater varied from 0.5 ng∙L^−1^ to 10 ng∙L^−1^ in the monthly sampling from 2015 to 2018, representing a very low risk to marine life during the 3 years of the interannual survey.

## Figures and Tables

**Table 1:** Total concentrations of 13 PAHs (ng∙L^−1^) in surface seawater from different locations [14,30,31,32,33,34].

Location	Survey Date	Concentrations (ng∙L^−1^)	References
Black Sea and Marmara Sea	2006–2007	605.3	[31]
Mediterranean Sea	2006–2007	306.1	[31]
Saronikos Gulf, Greece	2005–2006	245	[32]
Dalian, Northeast China	2010	79.57	[33]
Singapore	2005	10.84	[34]
Northwestern Japan Sea	2010	7.9	[14]
North Nanao Bay, Japan	2015–2018	1.67	This study

## Supplementary Materials

The following are available online at https://www.mdpi.com/1660-4601/17/3/904/s1, Table S1: Chrysene/pyrene ratio for various petrogenic sources. Table S2: PAHs ecological risk assessment guideline. Table S3: Toxic equivalent factor, negligible concentrations and maximum permitted concentrations for USEPA 16 priority PAHs.
