# Supplementary material for: Interannual Survey on Polycyclic Aromatic Hydrocarbons (PAHs) in Seawater of North Nanao Bay, Ishikawa, Japan, from 2015 to 2018: Sources, Pathways and Ecological Risk Assessment
Source: Int J Environ Res Public Health. 2020 Feb 1;17(3):904. doi: 10.3390/ijerph17030904 (PMC7038190; doi:10.3390/ijerph17030904)
Supplement: Supplementary file 1 [file ijerph-17-00904-s001.zip › Tables regarding ecological risk assessment.docx]

Supportive information

Interannual survey on polycyclic aromatic hydrocarbons (PAHs) in seawater of North Nanao Bay, Ishikawa, Japan, from 2015 to 2018: Sources, pathways and ecological risk assessment

Rodrigo Mundo^1^, Tetsuya Matsunaka^1,2^*, Hisanori Iwai^2^, Shouzo Ogiso^3^, Nobuo Suzuki^3^, Ning Tang ^4^, Kazuichi Hayakawa^2^ and Seiya Nagao^1,2^

^1^ Division of Material Chemistry, Graduate School of Natural Science and Technology, Kanazawa University, Kanazawa 920-1192, Japan; [rodrigomundo12@gmail.com](mailto:rodrigomundo12@gmail.com) (R.M.); [seiya-nagao@se.kanazawa-u.ac.jp](mailto:seiya-nagao@se.kanazawa-u.ac.jp) (S.N.);

^2^ Low Level Radioactivity Laboratory, Institute of Nature and Environmental Technology, Kanazawa University, Nomi 923-1224, Japan. [matsunaka@se.kanazawa-u.ac.jp](mailto:matsunaka@se.kanazawa-u.ac.jp) (T.M.), [h-iwai@se.kanazawa-u.ac.jp](mailto:h-iwai@se.kanazawa-u.ac.jp) (H. I.); [hayakawa@p.kanazawa-u.ac.jp](mailto:hayakawa@p.kanazawa-u.ac.jp) (K. H.).

^3^ Noto Marine Laboratory, Institute of Nature and Environmental Technology, Kanazawa University, Noto Cho 927-0553, Japan. [nobuos@staff.kanazawa-u.ac.jp](mailto:nobuos@staff.kanazawa-u.ac.jp) (N. S.), [shozoogiso@se.kanazawa-u.ac.jp](mailto:shozoogiso@se.kanazawa-u.ac.jp) (S. O.)

^4^ Institute of Medical, Pharmaceutical and Health Science, Kanazawa University, Kanazawa 920-1192, Japan. [n_tang@staff.kanazawa-u.ac.jp](mailto:n_tang@staff.kanazawa-u.ac.jp) (N. T.)

***** Correspondence: [matsunaka@se.kanazawa-u.ac.jp](mailto:matsunaka@se.kanazawa-u.ac.jp), Tel.:+81-76-151-4440 (T.M.)

Abbreviations list:

US EPA: United States Environmental Protection Agency

PAHs: polycyclic aromatic hydrocarbons

HPLC: High performance liquid chromatography

FLD: fluorescence detector

Nap: naphtalene

Ace: acenaphthene

Fle: fluorene

Ant: anthracene

Phe: phenanthrene

Flu: fluoranthene

Pyr: pyrene

BaA: benzo[a]anthracene

Chr: chrysene

BbF: benzo[b]fluoranthene

BkF: benzo[k]fluoranthene

BaP: benzo[a]pyrene

BPe: benzo[ghi]perylene

IDP: indeno[1,2,3-cd]pyrene

DBA: dibenzo[a,h]anthracene

Ex/Em: wavelength pares of excitation and emission

LOD: Limit of detection

RQ: Risk quotients

QV: quality value

NC: negligible concentration

MPC: maximum permissible concentration

TEF: toxic equivalent factors

**Table S1.** Chrysene/pyrene ratio for various petrogenic sources [39–42].

| Source name | Number of samples | Chrysene/Pyrene ratio (mean)α | Reference |
| --- | --- | --- | --- |
| Unburnt gasoline | 6 | 0.0775 | [45] |
| Gasoline engine exhaust | 3 | 0.141 | [43] |
| Unburnt diesel | 8 | 0.178 | [44] |
| Un used motor oil (gasoline car) | 10 | 0.335 | [42] |
| Used motor oil (gasoline cars) | 1 | 0.518 | [42] |
| Used motor oil (diesel cars) | 10 | 0.855 | [42] |
| Used motor oil (diesel busses) | 10 | 0.969 | [42] |
| Used motor oil (diesel trucks) | 10 | 1.38 | [42] |
| Diesel engine exhaust | 4 | 1.728 | [43] |
| Lubricating oil (re-refined oil) | 8 | 2.52 | [42] |

αMean values of the corresponding number of samples.

| For Individual PAHs | | | For ∑PAHs | | |
| --- | --- | --- | --- | --- | --- |
|  | RQ_(NCs)_^α^ | RQ_(MPCs)_^β^ |  | RQ_∑PAHs (NCs)_^γ^ | RQ_∑PAHs (MPCs)_^δ^ |
| Very low risk | 0 |  | Very low risk | ～0 |  |
|  |  |  | Low-risk | ≧1;<800 | 0 |
| Moderate-risk | ≧1 | <1 | Moderate-risk 1 | ≧800 | 0 |
|  |  |  | Moderate-risk2 | <800 | ≧1 |
| High-risk |  | ≦1 | High-risk | ≧800 | ≧1 |

**Table** S2. PAHs ecological risk assessment guideline [27].

^α^ The quality value of the negligible concentrations for individual PAHs

^β^ The quality value for the maximum permissible concentrations for individual PAHs

^γ^ The total quality value of negligible concentrations for 13 PAHs targeted in this study

^δ^ The total quality value of the maximum permissible concentrations for 13 PAHs targeted in this study

**Table** S3. Toxic equivalent factor, negligible concentrations and maximum permitted concentrations for USEPA 16 priority PAHs [25].

|  |  | Water (ng L^−1^) | |
| --- | --- | --- | --- |
| PAHs | TEFs^α^ | NCs^β^ | MPCs^γ^ |
| Naphthalene | 0.001 | 12 | 1200 |
| Acenaphthene | 0.001 | 3 | 300 |
| Fluorene | 0.001 | 3 | 300 |
| Phenanthrene | 0.001 | 3 | 300 |
| Anthracene | 0.01 | 0.7 | 70 |
| Fluoranthene | 0.001 | 3 | 300 |
| Pyrene | 0.001 | 0.7 | 70 |
| Benzo[a]anthracene | 0.1 | 0.1 | 10 |
| Chrysene | 0.01 | 3.4 | 340 |
| Benzo[b]fluoranthene | 0.1 | 0.4 | 40 |
| Benzo[k]fluoranthene | 0.1 | 0.4 | 40 |
| Benzo[a]pyrene | 1 | 0.5 | 50 |
| Dibenzo [a, h] anthracene | 1 | 0.5 | 50 |
| Benzo[ghi]perylene | 0.01 | 0.3 | 30 |
| Indeno[1,2,2-cd] pyrene | 0.01 | 0.3 | 30 |
| ∑PAHs |  | 31 | 3100 |

^α^ Toxic equivalent factors

^β^ The negligible concentrations for individual PAHs

^γ^ The maximum permissible concentrations for individual PAHs
